# Catheter-directed Angioembolization of Metastatic Melanoma of the Scalp: A Case Report

**DOI:** 10.51894/001c.144556

**Published:** 2025-09-30

**Authors:** C. Alex Matisse

**Affiliations:** 1 College of Osteopathic Medicine Michigan State University

## 100

### INTRODUCTION

Melanoma is a highly aggressive skin cancer arising from melanocytes, characterized by a high mutational burden primarily driven by ultraviolet (UV) radiation exposure. It progresses through local invasion, vascular dissemination, and metastasis to distant tissues. Scalp metastases are particularly concerning due to their aggressive nature and delayed diagnosis. Early detection and surgical excision of primary melanoma are critical for improving prognosis.

### CASE DESCRIPTION

A 53-year-old male presented with a massive malignant melanoma mass of the frontal scalp causing intractable hemorrhage. The initial diagnosis of melanoma in 2021 was treated with surgical resection, but subsequently recurred with metastasis. The patient had seen numerous oncologists and had intolerable side effects to chemotherapy, and chose not to pursue further chemotherapy at that time.

On presentation, the mass was necrotic, purulent, and malodorous, with hemorrhage requiring continuous compression dressings. Prior imaging included a CT angiogram which revealed the vascular supply to the tumor arises from the bilateral superficial temporal arteries. Interventional radiology was consulted for catheter-directed angio embolization for palliative treatment of the intractable bleeding. The bleeding was predominately on the right frontal aspect of the tumor. The catheter-directed angiography redemonstrated a large hypervascular scalp mass supplied from the superficial temporal arteries (right greater than left). The patient underwent uncomplicated superselective Gelfoam embolization of the right superficial temporal artery supplying the majority of the tumor (embolization of the left superficial temporal artery was not performed to reduce the risk of scalp necrosis). The patient responded well to embolization with cessation of bleeding within the same day.

### DISCUSSION/CONCLUSION

First-line treatments for metastatic melanoma include immune checkpoint inhibitors such as nivolumab and pembrolizumab, with BRAF or MEK inhibitors considered based on molecular markers. For patients who cannot tolerate these therapies or present with intractable symptoms, embolization can provide effective palliative care. This case demonstrates the role of embolization in controlling tumor-related hemorrhage and underscores its potential as an adjunctive therapy in metastatic melanoma management, particularly in cutaneous and hepatic metastases.

